# Reduced salinity tolerance in the Arctic grayling (*Thymallus arcticus*) is associated with rapid development of a gill interlamellar cell mass: implications of high-saline spills on native freshwater salmonids

**DOI:** 10.1093/conphys/cow010

**Published:** 2016-03-23

**Authors:** Salvatore D. Blair, Derrick Matheson, Yuhe He, Greg G. Goss

**Affiliations:** Department of Biological Sciences, University of Alberta, Edmonton, Alberta, Canada T6G 2E9

**Keywords:** Gill plasticity, osmoregulation, salinity, sodium–potassium ATPase, *Thymallus arcticus*

## Abstract

Arctic grayling (*Thymallus arcticus*) are salmonids that have a strict freshwater existence in post-glacial North America. Oil and gas development is associated with production of high volumes of hypersaline water. With planned industrial expansion into northern areas of Canada and the USA that directly overlap grayling habitat, the threat of accidental saline water release poses a significant risk. Despite this, we understand little about the responses of grayling to hypersaline waters. We compared the physiological responses and survivability of Arctic grayling and rainbow trout (*Oncorhynchus mykiss*) to tolerate an acute transfer to higher saline waters. Arctic grayling and rainbow trout were placed directly into 17 ppt salinity and sampled at 24 and 96 h along with control animals in freshwater at 24 h. Serum sodium, chloride and osmolality levels increased significantly in grayling at both 24 and 96 h time points, whereas trout were able to compensate for the osmoregulatory disturbance by 96 h. Sodium–potassium ATPase mRNA expression responses to salinity were also compared, demonstrating the inability of the grayling to up-regulate the seawater isoform *nkaα1b*. Our results demonstrated a substantially lower salinity tolerance in grayling. We also found a significant salinity-induced morphological gill remodelling by Arctic grayling, as demonstrated by the rapid growth of an interlamellar cell mass by 24 h that persisted at 96 h. We visualized and quantified the appearance of the interlamellar cell mass as a response to high salinity, although the functional significance remains to be understood fully. Compared with rainbow trout, which are used as an environmental regulatory species, Arctic grayling are unable to compensate for the osmotic stressors that would result from a highly saline produced water spill. Given these new data, collaboration between fisheries and the oil and gas industry will be vital in the long-term conservation strategies with regard to the Arctic grayling in their native habitat.

## Abstract (precis)

Arctic grayling were exposed to water conditions similar to industrial saline water spills. Grayling demonstrate a greatly diminished tolerance to seawater compared to euryhaline rainbow trout. Development of a novel response, a gill interlamellar cell mass, occurred within 24 hours in response to 17ppt (50% seawater) exposure.

## Introduction

The oil and gas industry is a socio-economic driver for both Canada and the USA, significantly impacting the overall gross domestic product of both countries (Honarvar *et al*., 2011). Natural gas has been pushed as a transition fuel to replace coal and reduce greenhouse gas emissions (Pacala and Socolow, 2004; Burnham *et al*., 2012). Consequently, pressure is mounting to develop deep shale reserves in northern areas, including Canada (Alberta, British Columbia, Northwest and Yukon Territories) and Russia (Grace and Hart, 1986; BP, 2015; Economides and Wood, 2009; Melikoglu, 2014). Oil and gas extraction technology (e.g. hydraulic fracturing, *in situ* development) results in large volumes of highly saline (up to 300 ppt; 10 times seawater) and organic contaminated flowback wastewater, with up to a 600-fold increase in [Na^+^] compared with local lakes and rivers (Allen, 2008). One of the clear risks of this industry is the potential for accidental release of these highly saline waters through a spill occurring at an on-site location or during transport to disposal wells (e.g. pipeline or trucking). Although oil and gas companies are taking measures, including contamination avoidance, water use and management, and reclamation (CAPP, 2015); spills are still common, especially involving incidental releases of hypersaline water resulting from hydraulic fracturing (Gross *et al*., 2013; Vengosh *et al*., 2014). Indeed, in a recent review of hydraulic practices, Goss *et al*. (2015) document a total of 113 spills of saline flowback fluid into flowing waters in the period 2005–2012 alone. Unfortunately, these resource-rich geographical areas directly overlap with the habitat for threatened native freshwater salmonids [Arctic grayling, mountain whitefish, bull trout, westslope cutthroat trout, and inconnu (Northcote, 1995; Rieman *et al*., 1997; McPhail and Troffe, 1998; Howland *et al*., 2001; Walker, 2005; Costello, 2006; Rodtka, 2009)]. A sudden influx of highly saline water into a stream would pose an immediate osmoregulatory perturbation for aquatic organisms, and it is essential to understand the potential impacts on these native species, especially given that most regulatory guidelines are routinely based on the responses of the euryhaline rainbow trout (Environment Canada, 1990; USEPA, 2002).

The process of osmoregulation is vital for all organisms, whereby animals must maintain a relatively constant internal concentration of ions irrespective of the external environment (Evans *et al*., 2005). In freshwater, fish are required to compensate for the loss of salts (Na^+^ and Cl^−^) by active salt absorption across the gills against the gradient presented by the hypotonic environment they inhabit. In contrast, in hypertonic marine environments, salts are excreted across the gills (Dymowska *et al*., 2012). Euryhalinity is defined by the ability to osmoregulate across a wide salinity range and characterizes some members of the Salmonidae family. Sodium–potassium ATPase (NKA) plays an important role in maintaining systemic and cellular ionic homeostasis, and its various isoforms have been identified to have a strong influence on the ability to tolerate seawater [*nkaα1a* as the freshwater isoform and *nkaα1b* as the seawater isoform (Richards *et al*., 2003; Bystriansky *et al*., 2006; Nilsen *et al*., 2007; Bystriansky and Schulte, 2011)]. Indeed, a recent phylogenetic analysis of the Salmonidae family has identified *nkaα1a* and *nkaα1b* sequences in all three subfamilies: Salmoninae, Coregoninae and Thymallinae (salmon and trout, whitefish and grayling, respectively; Dalziel *et al*., 2014).

Arctic grayling (*Thymallus arcticus*) are a native salmonid species that inhabit freshwater environments in post-glacial North America and, unlike other salmonids, do not undergo smoltification (Stamford and Taylor, 2004). Grayling inhabit lakes and rivers in the northern regions of Canada that directly overlap with large oil and gas reserves. Current status reports indicate that Alberta Arctic grayling populations are in serious decline and, as of 2015, a province-wide zero limit regulation has been implemented, requiring all grayling fishing to be catch-and-release only (AEP, 2015). Given the limited amount of understanding of the physiology of the Arctic grayling, the risks imposed and the threatened status, it is imperative to examine the salinity tolerance limits for grayling to ensure proper conservation strategies for this important native species. The objective of this study was to evaluate the physiological responses of the Arctic grayling to acute higher saline exposure. We compared the response with that of the euryhaline rainbow trout (*Oncorhynchus mykiss*) because trout are currently used for setting regulatory limits and remediation strategies. We hypothesized that Arctic grayling would demonstrate a lower salinity tolerance when exposed to short-term highly saline waters, as demonstrated by an inability to compensate for a similar osmotic stress compared with the rainbow trout.

## Materials and methods

### Animal collection

In collaboration with Alberta Environment and Parks, Arctic grayling (*Thymallus arcticus*) were collected via angling (fly-fishing) from Marten Creek, Alberta. Grayling were transported from Marten Creek to the University of Alberta's bio-secure aquatics facility using an Alberta Environment and Parks hatchery truck carrying a 1000 l tank containing oxygenated river water (∼9.76 mg O_2_ l^−1^, ∼20.4°C), chilled with ice bags. Fish were transferred from the truck and placed into a main holding tank (825 l) with aerated flow-through dechlorinated Edmonton tap water at 10°C. Grayling (20.6 ± 0.4 cm, 69.5 ± 3.6 g, means ± SEM) were maintained and allowed to acclimate in captivity for 2 months prior to experimentation. Rainbow trout (21.5 ± 0.4 cm, 111.8 ± 5.8 g) were reared to size in house from embryos generously donated by the Allison Creek Fish Hatchery (Alberta) and maintained in tanks supplied with the same facility water as the Arctic grayling. Trout and Arctic grayling were fed daily with crushed trout chow (Nu-Way^®^; Hi-Pro Feeds, Okotoks, AB, Canada). Grayling were supplemented with thawed *Artemia* sp. three times per week. Fish were fasted for 4–5 days prior to salinity exposure experimentation.

### Salinity exposures and sampling

Preliminary testing of exposures of grayling and trout to higher salinities was necessary to achieve a starting threshold salinity concentration and time point allowing blood parameters to be measured experimentally. A concentration of 50% seawater (17 ppt) with a 96 h time course was chosen because this was the highest concentration tolerated by grayling for a 96 h exposure. Identical recirculating systems (180 l) containing either freshwater (0–0.1 ppt) or 17 ppt saline water were constructed and chilled to 10°C. The saline water was produced by dissolving a salt mixture (Instant Ocean) in dechlorinated Edmonton tap water to 17 ppt. Salinity was measured daily by both a hand-held refractometer (Atago) and digital salinity probe (YSI Model 85; Yellow Springs, OH, USA) and maintained by addition of freshwater or artificial saline water at 17 ppt. Fasted fish were acutely transferred to experimental tanks (0 or 17 ppt saline water). Lethal sampling (*n* = 8) of fish occurred in control conditions (freshwater) at 24 h, and in 17 ppt at 24 and 96 h exposure time points. Anaesthetized fish (MS-222 200 mg l^−1^ buffered with NaHCO_3_ 400 mg l^−1^, time <3 min) were weighed, their length was recorded, and blood (1 ml) was immediately sampled via caudal vein puncture using non-heparinized syringes. Blood was centrifuged (2 min at 12 000***g***) and the resulting serum frozen and stored at −80°C for later blood osmotic and ion analysis. The second and third gill arches were excised and either rapidly frozen in liquid nitrogen or processed for microscopy.

### Serum Na^+^, Cl^−^ and osmolality

Serum samples from grayling and rainbow trout were diluted to appropriate volume with ultrapure water for analysis of [Na^+^] via atomic absorption spectrophotometry (Model 3300; Perkin Elmer, Norwalk, CT, USA). Serum [Na^+^] was calculated in millimoles per litre against a NaCl standard curve. Analysis of serum [Cl^−^] was performed by direct analysis on a digital chloridometer (Labconco, Kansas City, MO, USA). Total serum osmolality was measured on a freezing point depression osmometer (Micro Osmette; Precision Systems).

### RNA extraction, complementary DNA synthesis and sodium–potassium ATPase isoform expression

Excised gills were frozen in liquid nitrogen and stored at −80°C for further RNA isolation. Gill filaments were ground in liquid nitrogen with a mortar and pestle on a bed of dry ice. The resulting powdered tissue (∼30 mg) was transferred to 1.5 ml Eppendorf tube containing 1 ml of TRIzol^®^, and RNA was subsequently extracted and isolated via the TRIzol Reagent method according to the manufacturer's protocol (Ambion, Life Technologies, Carlsbad, CA, USA). Total RNA was resuspended in 30 μl of nuclease-free water, quantified and validated for purity via spectrophotometry (NanoDrop, ND-1000; Thermo Fisher Scientific, DE, USA). Total RNA (3 μg) was treated to remove residual DNA (DNase I; Thermo Fisher Scientific, Burlington, ON, Canada). The RNA integrity was checked via gel electrophoresis and the 28S and 18S bands were visualized. Synthesis of complementary DNA (cDNA) from RNA template (1 μg) was performed using SuperScript III Reverse Transcriptase (Invitrogen) with a mix of oligo(dT) and random primers.

Quantitative real-time PCR was performed in order to measure the relative abundance of mRNA expression of *nka* isoforms among the grayling and trout exposed to salinity at 24 and 96 h. The annotated partial sequences for Arctic grayling *nkaα1a* and *nkaα1b* (Dalziel *et al*., 2014) share an 89.87% nucleotide identity, making common gene-specific primer design and subsequent qPCR challenging. To navigate this obstacle confidently, we used the RNase H-Dependent qPCR technique and, in doing so, designed rhqPCR primers for grayling and trout, which are blocked and cleavable by the addition of a specific enzyme, RNase H2, according to the manufacturer's protocol (Integrated DNA Technologies, Coralville, IA, USA; Dobosy *et al*., 2011). This technique allows for the detection of target genes differing by only a single nucleotide owing to the high specificity resulting from the enzyme cleavage of the blocked primer bound to the complementary sequence (Dobosy *et al*., 2011). Gene-specific primers for *nkaα1a* and *nkaα1b* were designed, in addition to *elongation factor 1α* (*ef1α*), which served as a house-keeping gene (Table 1). Forward and reverse primers for rainbow trout *ef1α* expression were as previously developed in our laboratory (Boyle *et al*., 2015) and were found to be 80 and 100% identical to the grayling *ef1α* target sequence, respectively, and furthermore, they were tested and *ef1α* expression was shown to be stable in grayling and trout regardless of the treatment. All qPCR was performed in a light cycling PCR machine (ABI Prism 7500 sequence detection system). Samples were run in triplicate, with final reaction volumes of 10 μl per well containing 2× SYBR green PCR mastermix (Thermo Fischer Scientific, Rockford, IL, USA), 300 nmol of each primer, 1:20 dilution of sample cDNA and 0.5 μl RNase H2 enzyme buffer mixture. The PCR reaction mix was denatured at 95°C for 2 min, followed by 40 thermal cycles of denaturation for 15 s at 95°C and annealing and extension for 1 min at 60°C. Dissociation curve analysis was performed after each amplification reaction to ensure that a single product was obtained. Primer efficiency curves were run with a random grayling and trout cDNA sample separately in a 4-fold dilution pattern to evaluate the replication efficiency for each species (Table 1). Samples were normalized relative to *ef1α*, compared within genes with control (freshwater) levels and analysed statistically with one-way ANOVA and Dunnett's multiple comparisons test (Prism 6; GraphPad Systems).

**Table 1:** Nucleotide sequences of quantitative real-time PCR primers designed for the RNase H-dependent PCR

Gene	Accession information	Quantitative PCR primer (5′–3′)	Amplicon length (bp)	Primer efficiency
Arctic grayling *nkaα1a*	Accession: KJ175158 GI: 645929871	Forward: GACGCCTCTTGGAATTGA**rA**ATTGC/3SpC3/ Reverse: CCAGAATGACGGAGAGGA**rT**TAAGG/3SpC3/	98	1.89
Arctic grayling *nkaα1b*	Accession: KJ175159 GI: 645929873	Forward: GTGGCTGGAGAGTCCAA**rG**CACCC/3SpC3/ Reverse: CGTTCTGGAAGGCTTCTTT**rC**AACTT/3SpC3/	133	1.86
Rainbow trout *nkaα1a*	Accession: AY319391.1 GI: 34812026	Forward: GCCTCTCGGAATTGAAATTGA**rT**CACTG/3SpC3/ Reverse: GGATGGCAGCCATCCATA**rG**CCCAA/3SpC3/	117	1.92
Rainbow trout *nkaα1b*	Accession: AY319390.1 GI: 34812024	Forward: AAAGAGATTGAGCACTTTATCCA**rC**ATCAG/3SpC3/ Reverse: GACAGCTTCCAGCCA**rG**CCATG/3SpC3/	107	1.95
*ef1α*	Accession: AF498320.1 GI: 20269865	Forward: CTGTTGCCTTTGTGCCCATC Reverse: CATCCCTTGAACCAGCCCAT	82	1.96

Bold type ‘**r(X)**’ represents the inserted RNA base. Gene accession information is from the National Center for Biotechnology Information, and primer efficiencies as well as subsequent amplicon lengths are given. Abbreviations: GI, GenInfo Identifier; bp, base pairs.

### Interlamellar cell mass measurements

Gills were excised from grayling and rainbow trout and fixed in a 4% paraformaldehyde solution in phosphate-buffered saline overnight at 4°C. Gills were washed in phosphate-buffered saline and underwent ethanol dehydration prior to paraffin wax embedding. Microtome sections of gill tissue (7 μM thick) were fixed onto Superfrost^®^ plus microscope slides (Thermo Fischer Scientific, Rockford, IL, USA) and were stained with haematoxylin and eosin and examined under light microscopy (Leica DM RXA). Digital images were collected from three randomly selected fish, and two filaments from each fish with 10 adjacent lamellae were imaged (QI Click, QImaging). ImageJ software (National Institutes of Health) was used to calculate the interlamellar cell mass (ILCM) to lamellar length ratio as previously described (Ong *et al*., 2007). The height of the ILCM was measured parallel to the total lamellar length, starting from the edge of the ILCM bordering the filament to the most distal edge of the ILCM from the filament. Ratios were calculated and compared among the three treatments for both grayling and trout and analysed via one-way ANOVA (Prism 6; Graphpad Systems).

## Results

### Survival

Arctic grayling proved to be unable to cope well upon acute exposure to higher saline waters. Owing to limited numbers of available animals, a preliminary set of exposures was conducted on a small number of fish to define the approximate salinity tolerance. Results indicated that short exposure to 50% (17 ppt) seawater for 0–96 h was not lethal (0% lethality), but the fish were clearly distressed as shown by abnormal swimming behaviour and periodic loss of equilibrium; additionally, 100% mortality (*n* = 2) occurred at 100 h after exposure. Higher salinity (25 ppt; 75% seawater) resulted in 100% mortality (*n* = 2) within 24 h. Therefore, a 50% seawater concentration (17 ppt) and a 96 h time point were chosen for final physiological analysis. Rainbow trout showed no mortality at either 50 or 75% seawater, and the strain of trout housed in our facility (supplied by Raven Brood Trout Station) has been known to survive these conditions indefinitely, with survivability similar to trout used by Bath and Eddy (1979).

### Serum [Na^+^]

**Figure 1:**
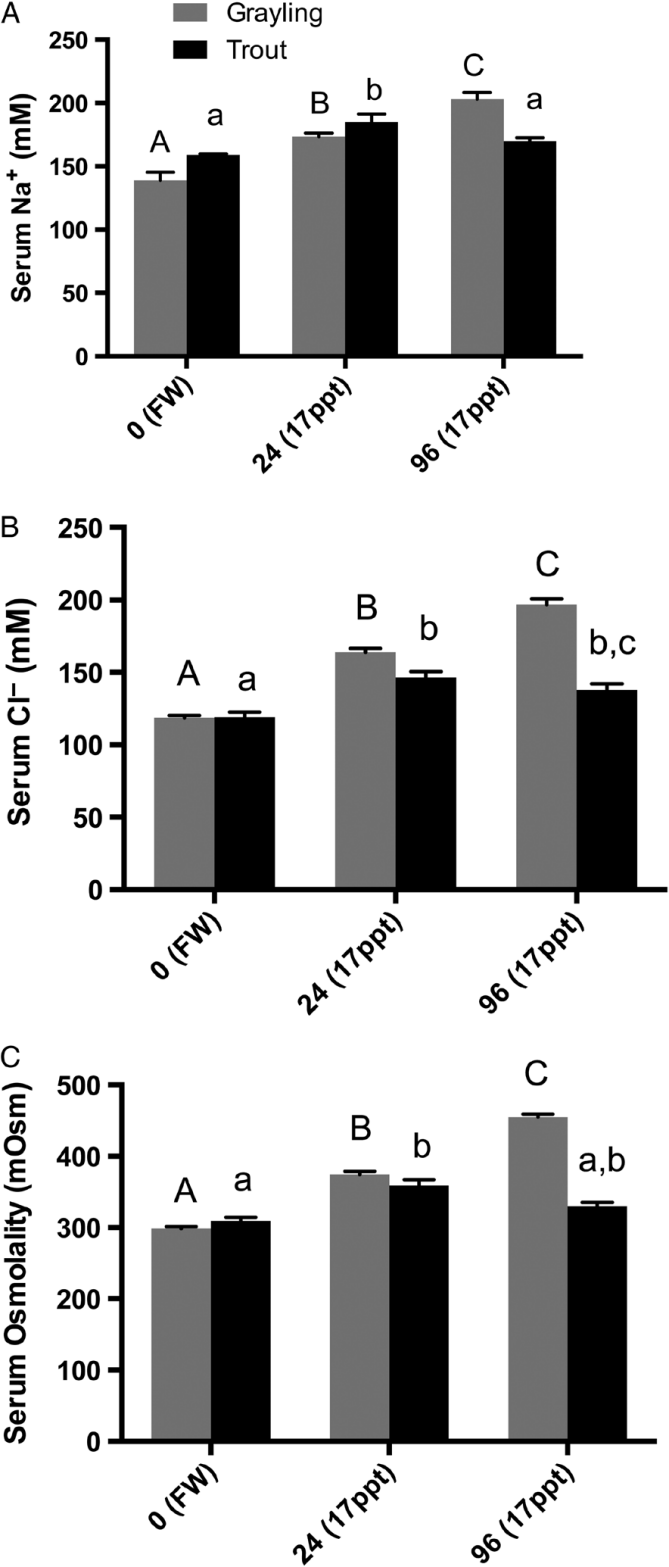
Serum sodium [Na^+^] (**A**), serum chloride [Cl^−^] (**B**) and serum osmolality (**C**) of Arctic grayling and rainbow trout exposed to freshwater (control; FW) and 17 ppt saline water at 24 and 96 h. Grayling and trout demonstrating significant increases in serum ions and osmolality at 24 h, and by 96 h serum concentrations were further elevated in grayling, but rainbow trout levels returned near to or were at control values. Grayling (grey bars) and rainbow trout (black bars) values are means + SEM, while dissimilar letters demonstrate significance using one-way ANOVA and Tukey's multiple comparisons. Capital letters denote grayling significance in comparison to grayling freshwater, whereas lowercase letters denote rainbow trout significance (*n* = 8, *P* < 0.05, one-way ANOVA).

The initial values of serum [Na^+^] of freshwater Arctic grayling were slightly lower, although not significantly, than those of rainbow trout (139 ± 6.5 vs. 159 ± 0.9 mM Na^+^, respectively). However, both grayling and trout demonstrated significantly elevated serum [Na^+^] by 24 h exposure, increasing by ∼25 and 16%, respectively, from baseline levels. However, by 96 h, trout serum [Na^+^] had returned to near control levels, whereas Arctic grayling demonstrated continued elevation in [Na^+^], reaching 203 ± 5.5 mM (Fig. 1A).

### Serum [Cl^−^]

Average values of serum [Cl^−^] for Arctic grayling and rainbow trout held in freshwater were nearly identical (118 ± 1.6 and 119 ± 3.4 mM Cl^−^, respectively). Serum [Cl^−^] increased significantly upon exposure to 50% seawater, increasing by ∼38% in grayling and ∼23% in trout after 24 h. At 96 h, average grayling serum [Cl^−^] was further elevated by 65% to 196.8 mM. The serum [Cl^−^] in trout returned closer to control values, falling from 146 mM Cl^−^ at 24 h to 138 mM at 96 h (Fig. 1B), although the [Cl^−^] was still statistically elevated at 96 h.

### Serum total osmolality

Total osmolality naturally followed a similar pattern to the [Na^+^] and [Cl^−^] during the exposures. Arctic grayling serum osmolality increased significantly from 299 mOsm in fish pre-exposure, to 374 mOsm after 24 h exposure to 50% seawater. A similar increase was demonstrated in the trout serum osmolality, increasing from 309 to 359 mOsm. The elevated total osmolality persisted in the grayling serum, reaching an average of 455 mOsm by 96 h; however, the trout serum osmolality significantly reduced back to control levels by 96 h in 50% seawater (Fig. 1C).

### Sodium–potassium ATPase gene expression

**Figure 2:**
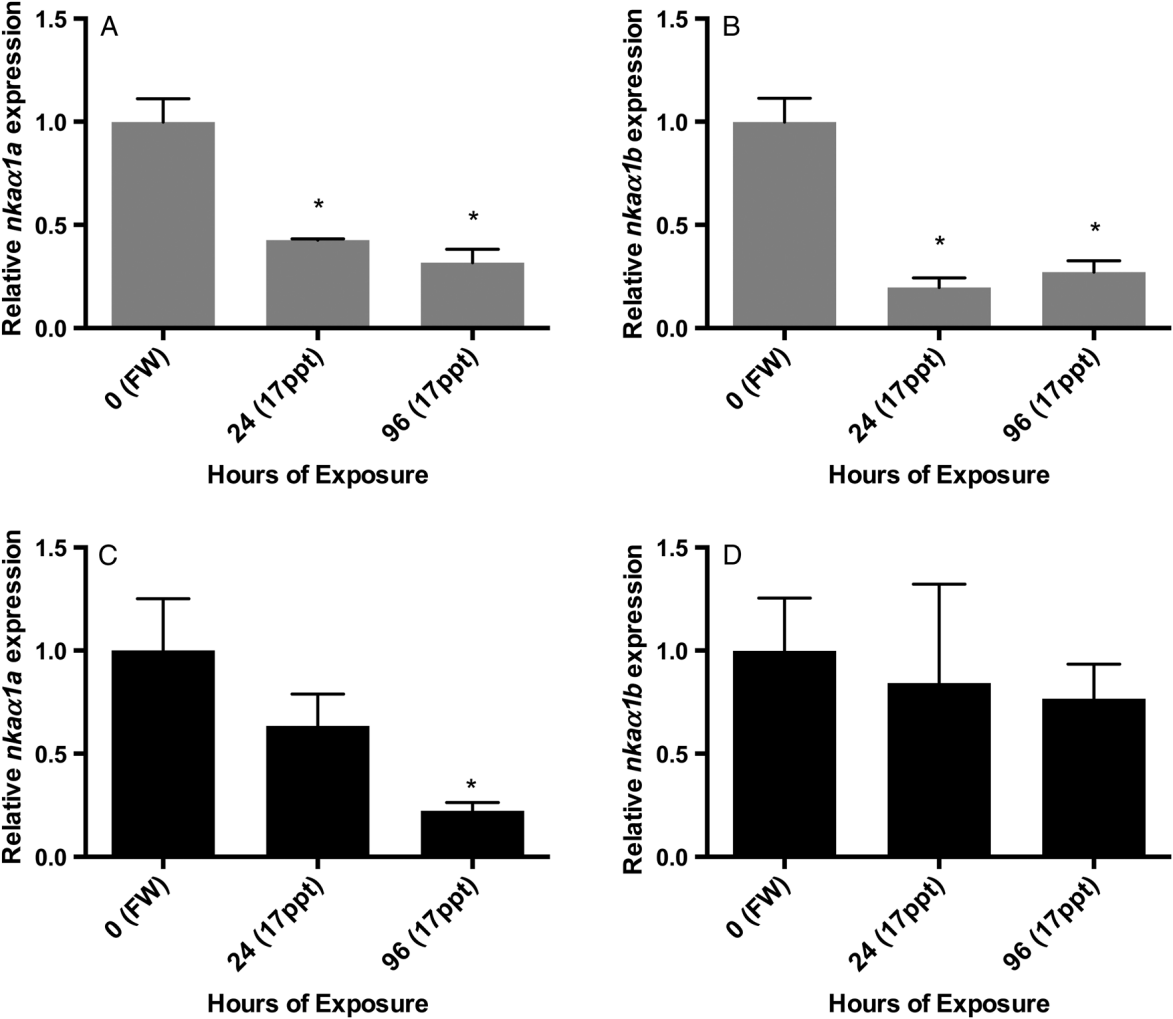
Gill expression of sodium–potassium ATPase isoforms *nkaα1a* and *nkaα1b* in Arctic graying (**A** and **B**) and rainbow trout (**C** and **D**) in freshwater (FW) and after 24 and 96 h exposure to 50% (17 ppt) seawater. Messenger RNA expression is normalized to the endogenous control gene *ef1α*, and significant differences from control freshwater levels are designated with an asterisk (*n* = 3, *P* < 0.05, one-way ANOVA, Dunnett's multiple comparisons test).

Gene expression data from qPCR analysis supported the expression of both isoforms of sodium–potassium ATPase, *nkaα1a* and *nkaα1b*, in all of the gill tissue samples analysed. Expression patterns for Arctic grayling indicated a significantly decreased mRNA expression of the freshwater isoform, *nkaα1a*, at 24 and 96 h when compared with control levels. A similar pattern was seen for the seawater isoform, *nkaα1b*, where it also demonstrated a significant decreased relative level of expression in the gills compared with control freshwater levels at 24 and 96 h (Fig. 2).

A slightly different pattern was seen through analysis of the rainbow trout gill *nka* gene expression. Upon exposure to 50% salinity, rainbow trout expression levels of *nkaα1a* mRNA showed a decreasing trend at 24 h, but expression was significantly lower than control levels at 96 h, similar to that of the grayling expression. In contrast to the grayling expression, gill *nkaα1b* levels in rainbow trout did not decrease significantly at either 24 or 96 h exposure to 50% salinity, but rather stayed similar to control freshwater expression (Fig. 2).

### Gill tissue plasticity

**Figure 3:**
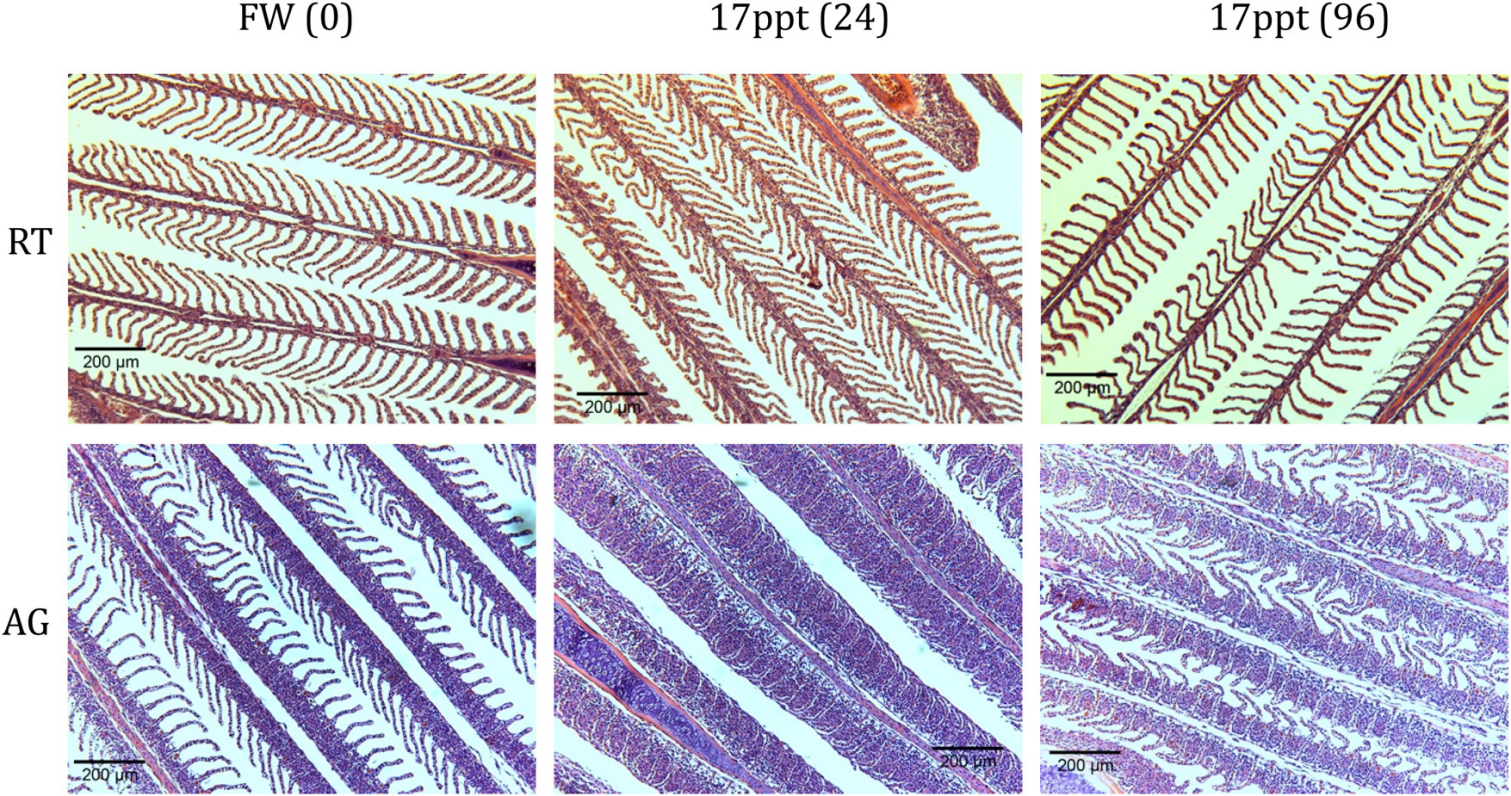
Light microscopy images showing comparison between rainbow trout (RT; top row) and Arctic grayling (AG; bottom row) gills in control freshwater (FW) conditions and after 24 and 96 h exposure to 50% seawater (17 ppt).

**Figure 4:**
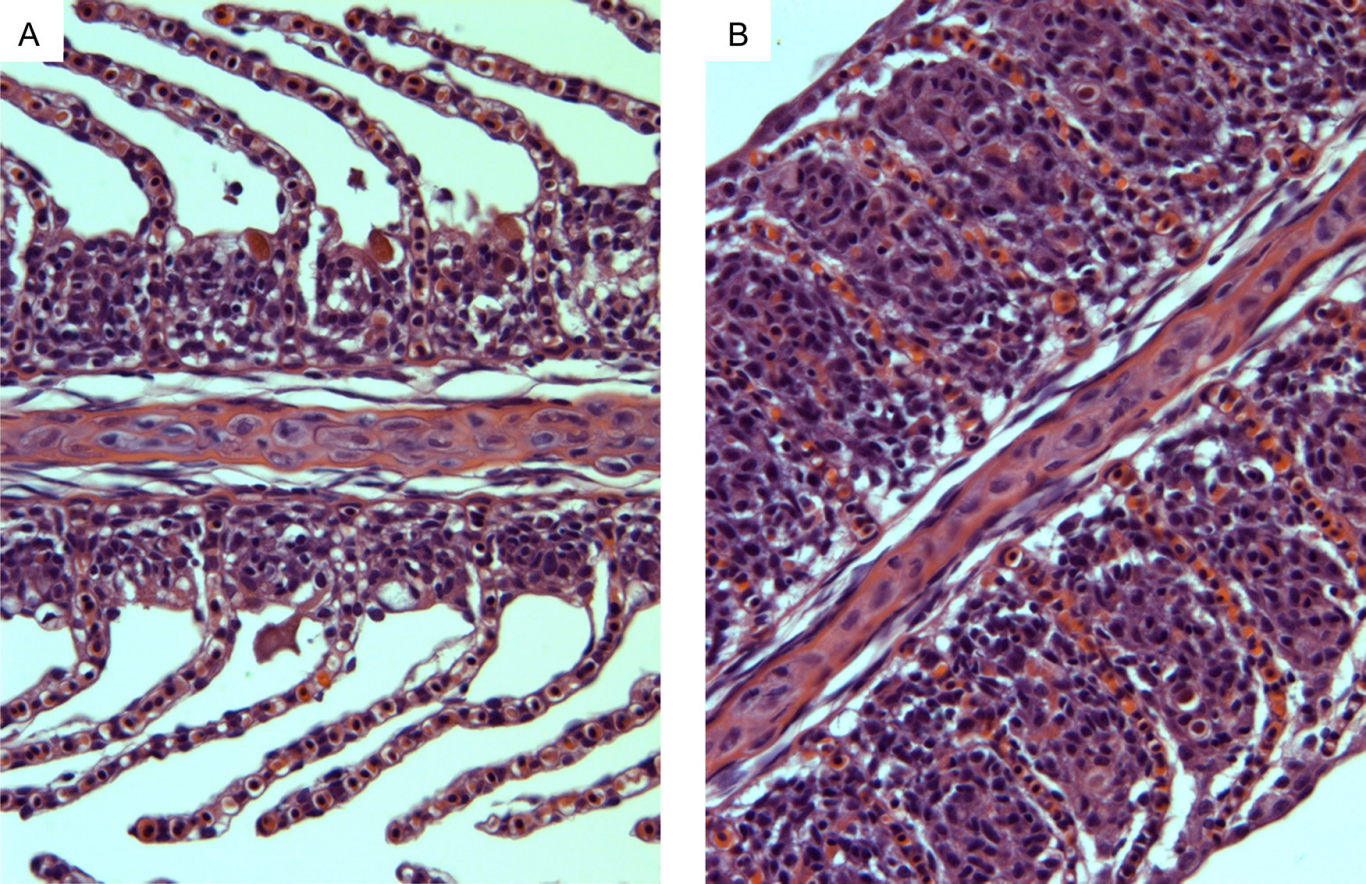
Light microscopy image showing increases in the interlamellar cell mass. Haematoxylin- and eosin-stained Arctic grayling gill filaments in freshwater (**A**) and after 24 h exposure to 50% seawater (17 ppt; **B**).

**Figure 5:**
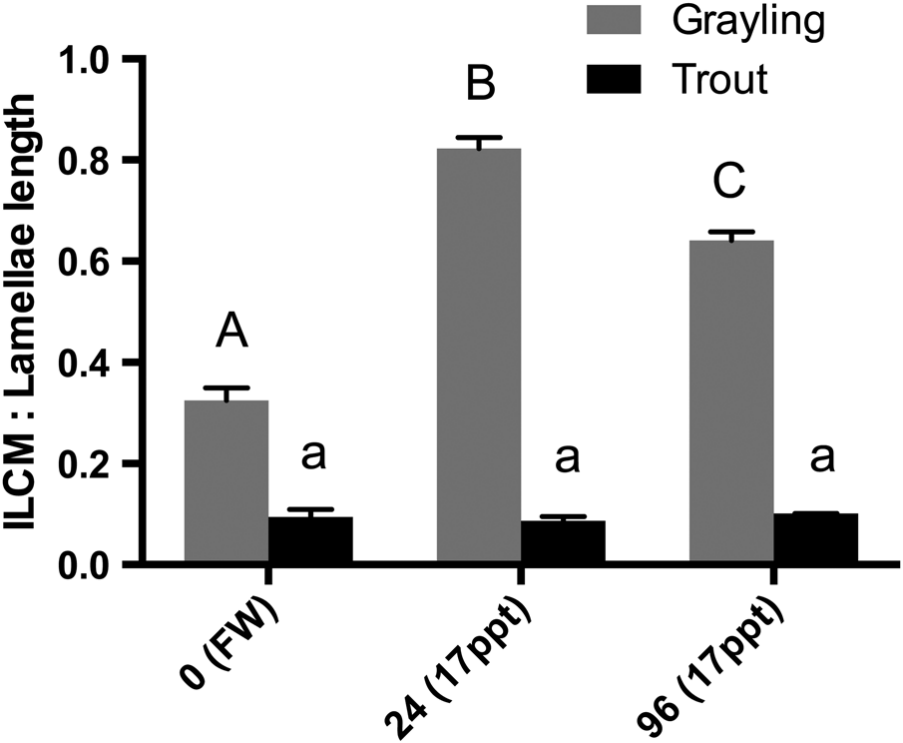
Measurements of the interlamellar cell mass (ILCM) of Arctic grayling and rainbow trout exposed to freshwater (FW; control) and 17 ppt saline water at 24 and 96 h. Grayling (grey bars) and rainbow trout (black bars) values indicate means + SEM, with significance demonstrated by dissimilar letters using one-way ANOVA. Capital letters denote grayling significance in comparison to grayling in freshwater, whereas lowercase letters denote rainbow trout significance (*n* = 3, *P* < 0.05, one-way ANOVA).

An intriguing result arose when comparing the morphology of the gills of these fish following exposure to 50% seawater at 24 and 96 h. Haematoxylin- and eosin-stained gill filaments exhibited a significant increase in the ILCM located at the base of the lamellae of the Arctic grayling (Figs 3 and 4). The height of the ILCM was quantified after exposure to 50% seawater for 24 h. We found a 156% increase in the ILCM of Arctic grayling (Fig. 5). This ILCM was not seen in the rainbow trout at any of the time points observed (Fig. 4). At 96 h, the grayling ILCM was still elevated 98% above control values, with a slight reduction from the 24 h peak (Fig. 5).

## Discussion

Research on salmonid salinity tolerance is extensive, because this family is most well known for anadromous fish species that naturally make the transition from fresh to saline water at some point in their life cycle (Morgan and Iwama, 1991; Staurnes *et al*., 1992; Singer *et al*., 2002; Richards *et al.*, 2003; Bystriansky *et al*., 2006; Larsen *et al*., 2008; Bystriansky and Schulte, 2011; McCormick *et al*., 2013; Dalziel *et al*., 2014). In this study, we aimed to investigate the effects salinity exposure may have on Artic grayling, a threatened salmonid species that has had a strict freshwater existence in North American for the past 3–5 million years (Stamford and Taylor, 2004).

Spills of saline process-affected water into aquatic and riparian areas have been documented across North America, and the possibility of saline spills into flowing water bodies containing Arctic grayling is increasing (Vengosh *et al*., 2014; USEPA, 2015; Goss *et al*., 2015). Current environmental testing for spill response, environmental impact assessment and remediation strategies use data from rainbow trout. For high concentrations of salts that commonly occur in the produced water (up to 10 times seawater or ∼300 ppt), even a 20-fold dilution of this water would still be above the salinity threshold of Arctic grayling if they were exposed to it for longer than 96 h. We have not yet investigated the 24 h salinity threshold for grayling, although our limited survivability data showed that animals (*n* = 2) exposed to 75% seawater in preliminary tests died before 24 h. Given a high-volume spill of 300 ppt produced water into a stream containing grayling, one would expect large amounts of death, because the osmotic stress would be the initial threat to these fish. In northern areas, fish in streams tend to congregate in deep overwintering holes because many sections of rivers and streams freeze completely in the winter. The severity of a spill of saline produced water at this time would be compounded by the high density of the fluid and the low stream flow, resulting in the settling of this water into overwintering pools and swamping of the fish, which are essentially ‘trapped’ for the winter months. Aspects of volume, concentration and time are important factors influencing the survivability of these fish given the occurrence of a spill and need further investigation. Our study clearly demonstrates that Arctic grayling have a much greater sensitivity to saline water exposure when compared with their euryhaline salmonid relative, the rainbow trout.

Arctic grayling were unable to osmoregulate successfully when exposed to even 50% seawater, as demonstrated by histological, physiological and molecular indicators. Grayling showed significant elevations of serum [Na^+^] and [Cl^−^] at 24 h, and these were even further elevated through the 96 h sampling period. This elevation of blood salinity would be detrimental for normal physiological function (Conte and Wagner, 1965; Jackson, 1981) and is likely to be the proximal cause of death owing to haematological failure.

Comparing rainbow trout and Arctic grayling, the responses in serum ion concentrations and osmolarity were similar in 50% seawater at 24 h. However, rainbow trout had the physiological capacity to overcome this stress and successfully reduced the ion load by 96 h, whereas grayling were unable to compensate. A salmonid comparison study by Bystriansky *et al*. (2006) demonstrated the differences in elevation patterns of plasma ions between three salmonid species acutely exposed to 32 ppt (∼100% seawater). Although some mortality did occur, all three species, Arctic char, Atlantic salmon and rainbow trout, were able to compensate for the osmotic disturbance and gradually lowered initial elevated plasma ion levels, with char being the least successful out of the group (Bystriansky *et al*., 2006). Likewise, Richards *et al*. (2003) showed that rainbow trout exposed to 40 and 80% seawater gradually decrease their initial ion elevations, returning to control freshwater levels by day 5. In contrast to these studies and the rainbow trout in the present study, Arctic grayling were unable to correct the salt load, and at 96 h the serum [Na^+^], [Cl^−^] and total osmolality were elevated significantly higher than both control and the already elevated 24 h levels. This pattern was also observed in landlocked Arctic char, which have limited sea water tolerance, whereby upon transfer to 100% seawater (32 ppt) the plasma osmolality levels rose from freshwater control levels and increased significantly until day 7 (Bystriansky *et al*., 2007).

Various studies have shown the link between salinity transfer and gill NKA expression and activity in salmonids (McCormick, 1996; Richards *et al*., 2003; Bystriansky *et al*., 2006; McCormick *et al*., 2013). In freshwater, NKA provides the driving energy for sodium absorption through mitochondrion-rich cells, whereas in seawater-adapted fish, NKA mediates the branchial excretion of sodium by maintaining the transepithelial electrical potential across the gill epithelium, allowing for the movement of Na^+^ from the blood back to the external environment through leaky junctions between mitochondrion-rich cells and accessory cells (Evans *et al*., 2005). In many instances, transfer of salmonids from freshwater to seawater results in the increased expression of *nkaα1b* mRNA and the decreased expression of *nkaα1a*, as seen in rainbow trout (Richards *et al*., 2003; Bystriansky *et al*., 2006), in Atlantic salmon smolts (McCormick *et al*., 2013), in brown trout (*nkaα1b* only; Larsen *et al*., 2008); in Arctic char and Atlantic salmon (Bystriansky *et al*., 2006). This differential expression pattern has been associated with the ability of salmonids to tolerate seawater, and in the present study both grayling and trout showed a significant decrease in expression of *nkaα1a* upon transfer to seawater, similar to that of previous findings. Following exposure to seawater, *nkaα1b* mRNA expression also showed a significant decrease in the grayling gill. Their apparent inability to up-regulate the *nkaα1b* isoform may indeed lead to the lack of salinity tolerance of grayling observed in this experiment. In support, Bystriansky *et al*. (2007) showed a similar pattern in a population of land-locked Arctic char, which upon exposure to seawater (32 ppt) displayed identical results to the grayling in our experiment, with both isoforms demonstrating decreasing levels of expression, and concluded that this was indicative of a loss of salinity tolerance. It is possible that the relative ratio of mitochondrion-rich cells to the total number of cells is altered during ILCM production, which might affect the result presented in Fig. 2, where relative expression to *ef1α* was demonstrated. However, Mitrovic *et al*. (2009), in a study using hypoxia and ILCM development in goldfish, demonstrated only minor changes in the number of mitochondrion-rich cells (<10%) per filament over 3 days. Furthermore, they demonstrated that the ionocytes migrated to the surface of the ILCM rather than increasing in number (Mitrovic *et al*., 2009). We are currently investigating the number of ionocytes in Arctic grayling associated with the ILCM.

Many studies use full-strength seawater (100% or 32–34 ppt) and longer acclimation periods (>7 days) to demonstrate changes in *nka* expression patterns. For example, Richards *et al.* (2003) demonstrated the down-regulation of *nkaα1a* and up-regulation of *nkaα1b* mRNA in the gills of rainbow trout exposed to 80% (∼26 ppt) seawater occurring at day 1 and persisting until day 5 following exposure. Upon exposure to 40% (∼13 ppt) seawater, rainbow trout showed decreased expression *nkaα1a*, but they did not demonstrate a significant increase in *nkaα1b* expression, and it was suggested that 40% seawater was insufficient to induce *nkaα1b* (Richards *et al*., 2003). This pattern supports our findings, where *nkaα1b* did not increase in 50% seawater, and suggests that there must be a ‘threshold cue’ for up-regulation of this seawater isoform to occur. In our study, grayling clearly had a substantial osmotic perturbation in their serum, but there was no upregulation of *nkaα1b*. Whether or not they have the capacity to do so or simply have not reached a required threshold in our study remains to be determined.

Histological images of the gill filaments revealed an impressive plasticity associated with the gill tissue of the Arctic grayling resulting from exposure to saline water. After 24 h exposure to 50% seawater, a significant increase in the ILCM of the grayling appeared, at times completely overgrowing the protruding lamellae and essentially eliminating the interlamellar region for water flow and decreasing the surface area of the gills dramatically. To our knowledge, the present study represents the first evidence of a saline-induced increase in the ILCM in any salmonid, and is intriguing with respect to the rapid time course (<24 h exposure) with which it appeared in the Arctic grayling gill.

Gill plasticity or an increase in the ILCM are not new observations; indeed, this has been well documented in the literature and can happen as a result of many factors. The classic example of gill remodelling occurs in crucian carp (*Carassius Carassius*) and goldfish (*Carassius auratus*) in response to hypoxia. In control animals, a thick ILCM exists, and this is rapidly reduced upon exposure to hypoxic conditions. This is proposed to create greater surface area for oxygen uptake (Sollid *et al*., 2003; Sollid and Nilsson, 2006; Nilsson *et al*., 2012). Decreases in the ILCM have also been documented in response to temperature variation in the goldfish when acclimated to 25°C compared with those acclimated to 7°C (Mitrovic and Perry, 2009). However, in response to high environmental ammonia (Sinha *et al*., 2014), crucian carp and goldfish increased their ILCM. Likewise, Wright and colleagues documented killifish (*Kryptolebias marmoratus*) increasing their ILCM in response to air exposure to prevent water loss (Ong *et al*., 2007; Turko *et al*., 2011). Wright's group also found that the ILCM of the killifish was decreased in seawater-acclimated animals compared with freshwater-acclimated fish (LeBlanc *et al*., 2010). The only documented increase in ILCM by a salmonid was observed in brook trout (*Salvelinus fontinalis*) in response to aluminum exposure in slightly acidic water (Mueller *et al*., 1991).

We suggest that this particular gill remodelling would be a beneficial defensive mechanism upon exposure to high salinity, limiting the uptake of salts (Na^+^/Cl^−^) and preventing water loss across the gill epithelium by reducing the exposed lamellar surface area. As the results indicate, the ILCM, although still highly elevated at 96 h, does become slightly more reduced than its maximal height at 24 h. We hypothesize that the decrease in ILCM from the 24 h point to the 96 h level may be in response to the grayling blood becoming hypercapnic or hypoxic as a result of reduced gas exchange attributed to the ILCM. Theoretically, the ability to increase ILCM would be advantageous to the grayling during a short-term spill of saline-produced water. However, for a longer-term spill or if faced with environmental hypoxia, the ILCM would have deleterious consequences.

We propose that the appearance of an ILCM may be able to serve as a biological marker of grayling exposed to highly saline water. Given the simplicity of field fixation of moribund animals, this has the potential to be developed as an indicator of saline exposure following saline spills into grayling-bearing waters. Development of the ILCM as a viable marker for exposure requires further research into the rate of ILCM formation, the persistence/reversibility after the saline exposure and the physiological consequences of the ILCM (i.e. blood O_2_ and CO_2_ concentrations).

In conclusion, the present study has demonstrated a significantly lower salinity tolerance by the Arctic grayling in comparison to the rainbow trout. From a conservation physiology perspective, these data reveal the high sensitivity of grayling to an environmental spill of produced water. Our study highlights the lethal effects on Arctic grayling exposed to saline water at 50% seawater (17 ppt). Owing to the non-anadromous ancestral life history and having a strictly freshwater existence for more than 3 million years in North America, we suggest that in these acute salinity conditions Arctic grayling have lost the ability to execute the necessary osmoregulatory mechanisms to cope with higher salinity environments.

As a main byproduct of hydraulic fracturing, highly saline produced water poses a great risk to freshwater habitats and the aquatic organisms. With the expansion of the oil and gas industry, there is an increased risk of spills of produced water. We must be aware of the conservation impact that spills may have on native salmonid species that cannot compensate physiologically for such osmoregulatory challenges. Investigations on the physiological responses to acute salinity of other native freshwater salmonids (bull trout, mountain whitefish and cutthroat trout) have not been conducted. Compared with rainbow trout, which are used as an environmental regulatory species, Arctic grayling are unable to compensate for the osmotic stressors that would result from a highly saline produced water spill. Given these new data, collaboration between fisheries and the oil and gas industry will be vital in the long-term conservation strategies with regard to the Arctic grayling in their native habitat. Increasing research aimed at the fisheries located in these industrially impacted regions will be critical in coming to informed conclusions on the sustainable coexistence of these combined resource interests.

## Funding

This research was supported by a Natural Sciences and Engineering Research Council (NSERC) Discovery Grant [203736] to G.G.G. We thank Alberta Environment and Parks for in-kind financial support in the grayling collection.
